# Music Therapy as a Topic in Medical Education: Course Concept and Student Evaluation of an Elective Course for Medical Students

**DOI:** 10.1177/23821205241234537

**Published:** 2024-02-22

**Authors:** Susann Kobus, Ursula Felderhoff-Mueser, Elke Lainka, Joachim Fandrey, Sven Benson

**Affiliations:** 1 Department of Paediatrics I, University Hospital, University of Duisburg-Essen, Essen, Germany; 2 Center of Artistic Therapy, University Medicine Essen, Essen, Germany; 3 Center for Translational Neuro- and Behavioural Sciences, C-TNBS, Faculty of Medicine, University Duisburg-Essen, Essen, Germany; 4 Clinic for Pediatrics II, University Children's Hospital Essen, University of Duisburg-Essen, Essen, Germany; 5 Institute of Physiology, University Hospital Essen, University of Duisburg-Essen, Essen, Germany; 6 Institute for Medical Education, University Hospital Essen, University of Duisburg-Essen, Essen, Germany

**Keywords:** music therapy, medical education, pediatrics, medical care, neonatology

## Abstract

**OBJECTIVES:**

Music therapy has been proven as a safe and well-established intervention in healthcare to relieve symptoms and improve quality of life. While music therapy is already established in several settings to supplement medical care, there is a lack of integration in the field of medical education.

**METHODS:**

We report on the implementation and evaluation of a teaching concept for a five-day-intensive-course on music therapy. The course was offered as an elective course for medical students at the University Duisburg-Essen. At the end of the course, students filled out a free text questionnaire to assess the students’ perception of the course, and additionally answered standardized questions by the structured EVALuna online evaluation tool of the University of Duisburg-Essen.

**RESULTS:**

All students (N = 35) who participated in the music therapy course between September 2019 and March 2023 completed the questionnaires and N = 21 students filled out the EVALuna. Most students (89%) chose the course because of their interest in alternative and supportive therapy options to improve patients’ well-being. About 46% had previous musical experience and passion and fun with music and 37% of the students were interested in the interdisciplinary academic subject that combined music and medicine. EVALuna online evaluation reflected high satisfaction with the course.

**CONCLUSION:**

Due to the well-proven effectiveness and evidence of music therapy as well as the positive perception of medical students, music therapy should be further established in medical care and medical education.

## Introduction

Non-pharmacological interventions such as music therapy have promising potential to complement traditional medical treatments and promote recovery and well-being. Music therapy is increasingly applied in the care of preterm infants, children, and adolescents in many countries worldwide.^1-3^ Music therapy is not only an art but also a science, a process of human interaction and a form of therapy with the medium music,^4^ which is used within a therapeutic relationship to address the physical, emotional, cognitive, and social needs of individuals.^5^ Music therapists are increasingly available in palliative care teams to promote the clinical services and to meet the holistic needs of the patients and their families.^6^ In music therapy, the patient and therapist actively engage in singing, improvising, and listening to music, depending on the patient's musical preferences. Individual musical experiences can develop within a therapeutic relationship based on individual assessment, treatment, and evaluation.^7^

Music therapy serves as a mean of interaction, as a non-verbal form of communication and to promote the patient's individual development.^8^ Numerous studies have examined the effects of music on somatic and mental illnesses.^9,10^ Live therapist-directed music therapy has a stabilizing effect on vital signs of preterm infants^11-14^ and their parents.^15,16^ It has positive effects on physiological functions in preterm infants during a hospital stay.^17-19^ Music therapy promotes social interactions in the hospital and helps children with cancer and their families to better cope with their illness.^20^ Beneficial effects of music therapy have also been documented in adults suffering from post-traumatic stress disorders,^3^ and in cancer patients.^21,22^ Compared to standard care, music therapy interventions have beneficial effects on pain, anxiety, depression,^23^ fatigue, and help to promote well-being in daily live and to increase a sense of control and hope.^24-27^ Music therapy can reduce impulsiveness and increase self-regulation^28,29^ and resilience in newly diagnosed cancer patients.^30^ These effects can be explained by underlying neurobiological mechanisms: Music that produces intense pleasure or excitement activates the neural systems of reward and emotion in a similar way to other biologically relevant stimuli.^31^ The ability of music to induce pleasure and to stimulate endogenous reward systems suggests that our mental and physical well-being greatly benefits from music.^31^ Music is not only beneficial to the well-being of patients, but also shows positive effects on activities and services of medical staff.^32^

Music therapy can be integrated in medical practice, complementing medical treatments, which makes it relevant and interesting for medical education.^33^ Music therapy combines medicine with music and psychology,^34^ which offers an insight into an area that is both scientifically and clinically relevant.

Since 2018, music therapy is offered as an elective course for medical students at the Medical Faculty of the University of Duisburg-Essen. The course does not train professional music therapists. It is an elective course for medical students to gain an expanded overview of complementary therapies such as music therapy with self-experiences. The self-perceptions in practical work with a qualified music therapist are more formative than the theoretical teaching by a music therapist.^35^ In addition to those experiences the medical students see the patients from a different perspective than a physician.^36^ This makes the University of Duisburg-Essen the only institution that has integrated the subject music therapy into conventional medical education in Germany.

We report on the implementation and evaluation of the course concept. Specifically, we have investigated the motivation to participate and the students’ perception of the music therapy elective course.

## Methods

### Study Design

The study was designed as a prospective local study on medical students of the elective course *Music therapy in pediatrics (children's hospital and neonatology)* at the University of Duisburg-Essen. During the course this questionnaire-based survey was conducted anonymously. The students received the questionnaire at the end of the intensive five-days-course and returned it to the lecturer in a closed envelope. In addition, the EVALuna online survey, a structured and anonymous evaluation tool implemented at the Medical Faculty of the University Duisburg-Essen, was used to evaluate students’ satisfaction with the course.^37^ The students received a link to take part in the online satisfaction survey, which could only be used once for this subject. This means that both data collections could take place anonymously.

The requirement of the ethical approval to conduct this study was waived by the local ethics committee of the Medical Faculty of the University of Duisburg-Essen.

### Sample and Procedure

All medical students at the University Duisburg-Essen who participated at the elective course *Music therapy in pediatrics (children's hospital and neonatology)* (Department of Paediatrics I, University Hospital, University of Duisburg-Essen, Essen, Germany) between September 2019 and March 2023 were invited to participate in the evaluation of the course. The students were informed that they were free to participate, and that they agreed to participate in the survey by returning the completed questionnaire. On the questionnaire, the students also agreed to take part in the evaluation of the EVALuna online survey. The completion of the questionnaire was considered as informed consent. Completing the EVALuna questionnaire is part of the standard evaluation process conducted for all courses at the Medical Faculty. There were no exclusion criteria for students to participate in the study. Because the elective course *Music therapy in pediatrics (children's hospital and neonatology)* is the only elective course in the field of complementary therapies at the University of Duisburg-Essen, there were no inclusion and exclusion criteria for the selection of the analyzed subject.

### Elective Course of Music Therapy

The study program *human medicine* at University of Duisburg-Essen is comprised of three parts. The first part (“pre-clinical stage”) includes the first four semesters, the second part (a clinical stage”) the semesters 5–10, and the final part (“Practical Year”) includes the semesters 11 and 12. During the clinical stage, students can choose one of sixty elective courses.

The major aim of the elective course *Music therapy in pediatrics (children's hospital and neonatology)* is an affective learning aim, i.e. that medical students learn to see the patient from a different perspective and, in addition to the usual, get cognitive approaches during medical studies, to have a closer view on patients’ emotions and experiences.

**Figure 1.**
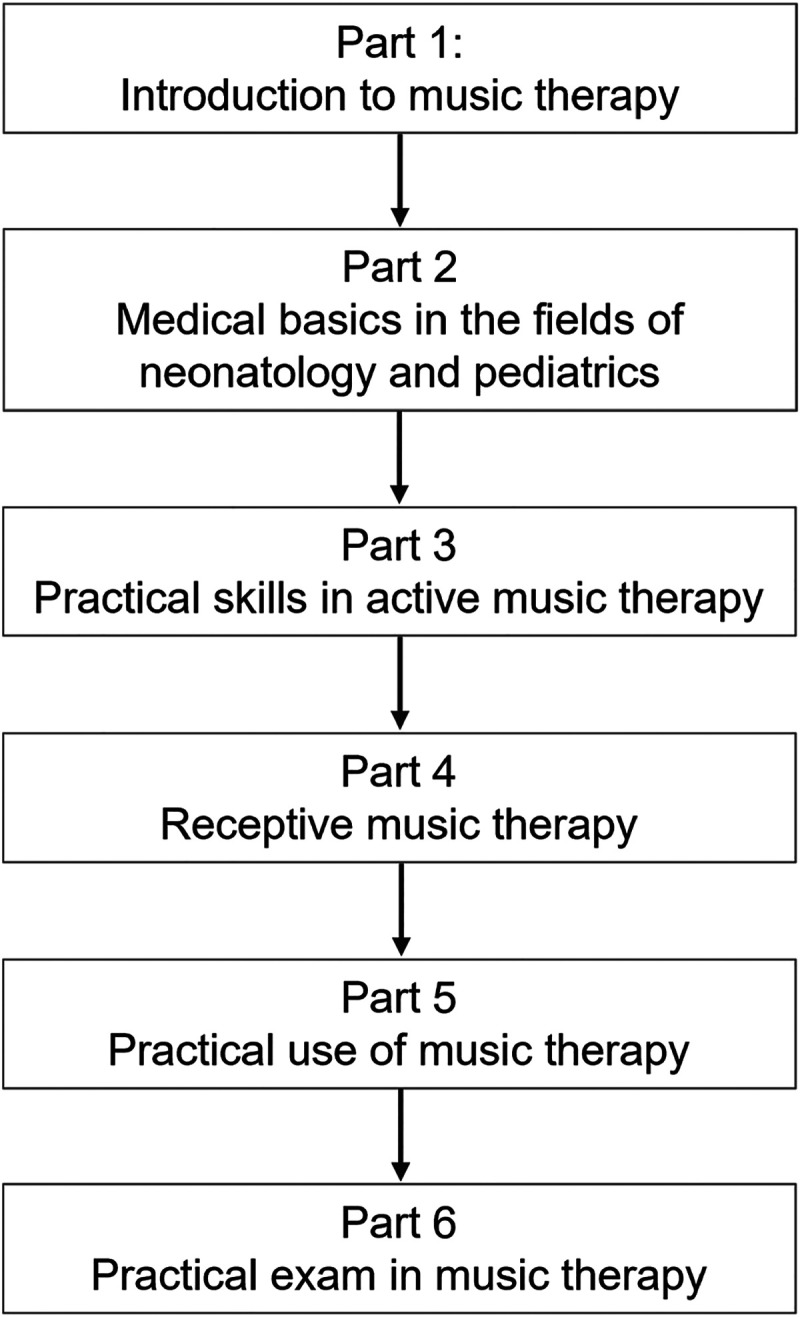
Curriculum of the music therapy course.

From the year 2018, the University of Duisburg-Essen is offering the special course *Music therapy in pediatrics (children's hospital and neonatology) (*Department of Paediatrics I, University Hospital, University of Duisburg-Essen, Essen, Germany*)* to provide the opportunity for medical students to gain insights about music therapy as an approach to supplement and support basic medical care. The course is offered once a semester every March and September for a maximum of 12 students. The course has six parts and takes place during five consecutive days (Figure 1).

Part 1: Introduction to music therapy

The first part provides an in-depth overview of music therapy directions, their theoretical background, origin, procedures, and the areas of application of music therapy. Music therapy is presented as a complementary therapy to medical care in inpatient and outpatient settings. Information about the applicable ethical principles of the profession, the legal framework and an overview of the existing national and international professional organizations are integrated. This part also serves to integrate the study group and to compensate for the different input skills of the students. An exchange of previous technical knowledge and musical experience of the students is analyzed.

Part 2: Medical basics in the fields of neonatology and pediatrics

This part provides an overview of basic medical knowledge in the areas of neonatology and pediatrics which are a relevant background for the elective course. This includes pediatric subspecialties such as ​​transplantation medicine for babies, children, and adolescents.

Part 3: Practical skills in active music therapy: Improvisation

The students comprehensively learn about the practical field of active music therapy with the various music therapy technical directions and the most important areas of application.

The focus of this part is making music without compositional requirements (improvisation) and the application in a musical-therapeutic relationship. Various percussion and therapeutic instruments such as sansula, ocean disc, zaphir, xylophones, tone bars, egg-shakers, vibraslaps, snare drum, tambourine, cymbals, triangle, maracas, chimes, cajons, bongos, a keyboard, and a guitar, are available for improvisation.

The major aim of this part is that students learn different forms of playing and conversation in the individual single and group music therapy sessions. Empathy and self-reflection are encouraged as essential prerequisites for therapeutic understanding and interaction. The students can understand their own and others’ experiences and behavior unbiasedly as indications of an overarching, biographical and reflecting structure. This part conveys an exercise in basic therapeutic skills such as role acceptance and role distance, empathy, group leadership and the improved perception of one's own subjectivity.

Part 4: Receptive music therapy

With the receptive forms of music therapy, the students learn further options to utilize music therapy. The students learn to play the instruments sansula, kalimba, ocean disc, zaphir or monochord and they are able to actually engage receptively to experience themselves. They learn to reflect the music. The initiation of guided imagery with music as relaxation exercises is also a theme of this part.

Part 5: Practical use of music therapy

As final step, the skills acquired in active and receptive music therapy are directly applied to patients in the patient room of the nephrology, gastroenterology, and neonatology wards at the University Children's Hospital Essen. Under the guidance and with the help of the lecturer, a qualified music therapist, the students prepare and direct own music therapy sessions for the patients with different diseases and different health status. Based on their own experiences and reflecting the group processes, the students experience the connection between musical and verbal expression and psychological structure. They become familiar with the appropriate preparation of a therapeutic or counseling relationship in music and language. For quality assurance and best practice, supervision as a reflection process is carried out by the lecturer with the students after the sessions.

Part 6: Practical exam in music therapy

**Figure 2.**
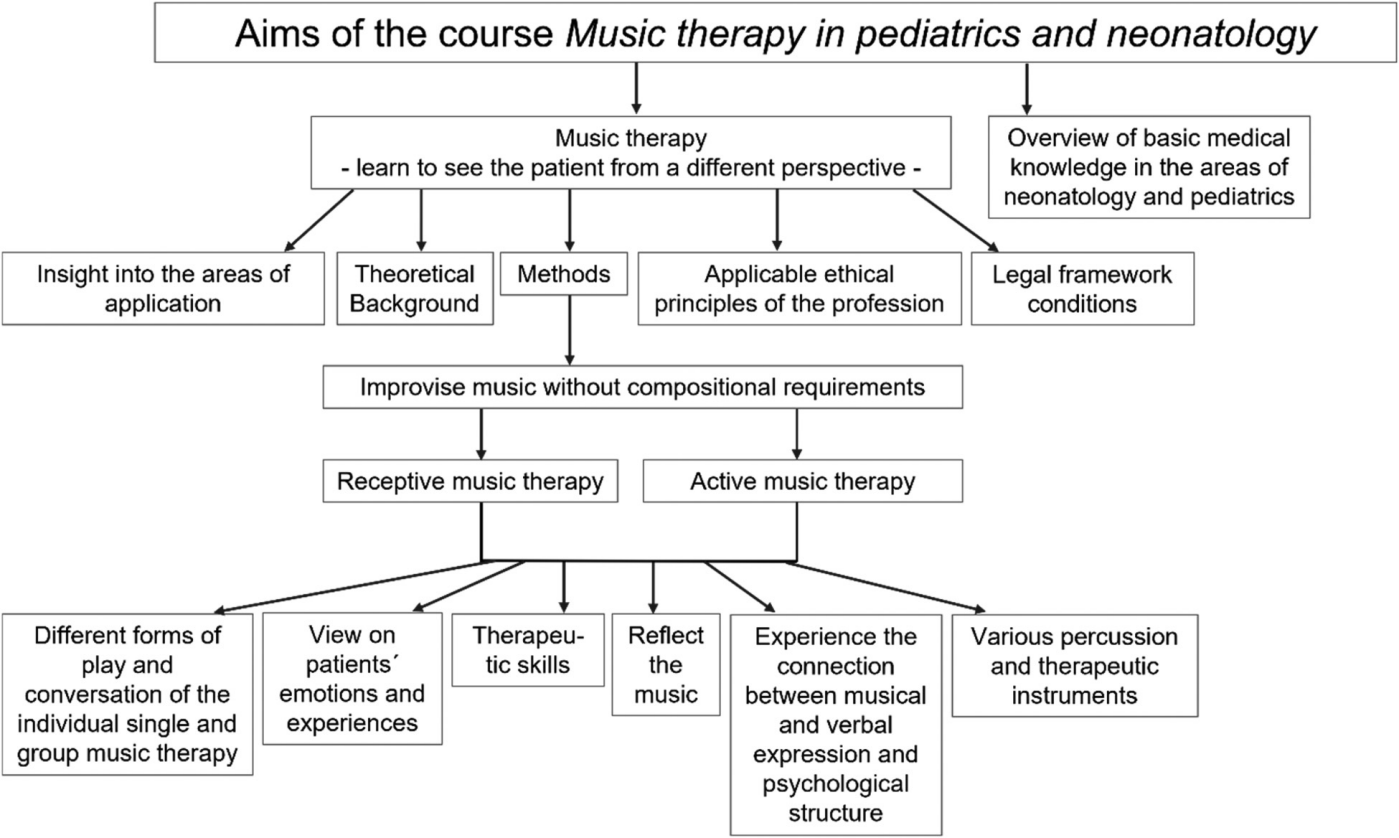
Aims of the music therapy course.

The students independently direct their own music therapy sessions on their own responsibility, but with the presence of the qualified music therapist, with the patients in the nephrology, gastroenterology, and neonatology wards in the University Children's Hospital Essen as an examination. The lecturer gives feedback and a graded diploma. Figure 2 presents an overview of the learning objectives.

### Questionnaire

At the beginning of the elective course *Music therapy in pediatrics (children's hospital and neonatology)*, the medical students were asked whether they had any prior knowledge of music therapy or whether they could imagine how music therapy would be applied to the patients. At the end of the course, the medical students filled out an anonymous questionnaire about how they had perceived the five days elective course of music therapy. The questionnaire consisted of four free text questions. Free text questions were used to provide participants the opportunity to respond at an individual amount, and not to restrict answers by closed questions.

- Why did you decide to take part at the course Music therapy in pediatrics (children's hospital and neonatology)?
- Did the course fulfill your expectations and wishes?
- Describe and evaluate your perceptions that you made during the music therapy course in the children's hospital and neonatology!
- What experiences and impressions will you take with you for your future?

### EVALuna Online Evaluation Tool

The EVALuna online evaluation tool of the Medical Faculty of the University Duisburg-Essen consisted of nine questions with a seven-point Likert scale, ranging from 7 = “absolutely satisfied” to 1 = “absolutely unsatisfied”. In detail, students were asked:

- Overall, how satisfied are you with this course?
- How satisfied are you with the performance of the lecturers?
- How satisfied are you with the exam situation of this course?
- How satisfied are you with the preparation for the exam during this course?
- How satisfied are you with the organization of this course?
- How satisfied are you with the content of this course?
- How satisfied are you with the general conditions of this course (rooms, technical equipment, etc)?
- How satisfied are you with the tools for the preparation and follow-up of this course (script, slides, etc)?
- How do you rate your subjective increase in knowledge through this course?

### Statistical Analyses

Descriptive analyses were conducted for sociodemographic and EVALuna data. The number of the medical students who participated in the music therapy course, their gender and their national origin are given in absolute frequencies and percentages. Continuous data (age, number of semesters, EVALuna data) are presented as mean values and ranges. The contents of the free text answers of the survey were categorized and summarized, and the term categories are reported as absolute frequencies and percentages. All graphics were created with Microsoft Excel 365.

## Results

### Students

Thirty-five medical students at the University Duisburg-Essen participated in the eight courses of *Music therapy in pediatrics (children's hospital and neonatology)* between September 2019 and March 2023. The students had a median age of 25 years (range 22–32) and 89% of these were born in Germany. On average, students attended the 12th semester of their medical education (Table 1).

**Table 1.** Characteristics of the medical students participating at the course of music therapy.

	All students (n = 35)	Female (n = 28)	Male (n = 7)
Age at participating (years), mean (range)	25 (22–32)	25 (22–32)	25 (22–30)
Born in Germany, n (%)	31 (89%)	24 (86%)	7 (100%)
Number of semesters of medical education, mean (range)	12 (10–18)	12 (10–18)	12 (10–16)

### Questionnaire

At the beginning of the elective course *Music therapy in pediatrics (children's hospital and neonatology)* 94% of the students had no prior knowledge about music therapy and could not imagine how music therapy is directed in an inpatient setting. After the music therapy course, we received completed questionnaires from all thirty-five students.

### Reasons for Choosing Music Therapy

**Figure 3.**
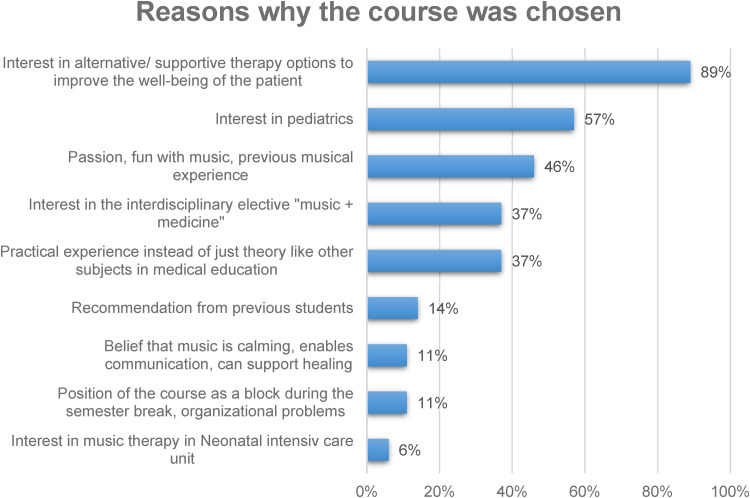
Reasons why the elective course *Music therapy in pediatrics (children's hospital and neonatology)* was chosen by students of medical education at University Duisburg-Essen.

The answers to the first question showed that most of the students (89%) were interested in alternative and supportive therapy options to improve the well-being of the patients. About 57% of the students in medical education have chosen the music therapy course because of their interest in pediatrics. We also had students (46%) who had previous musical experience and passion and fun with music. About 37% of the students said that they were interested in the interdisciplinary academic subject, where music and medicine are combined. Also 37% of the students preferred the subject because it is practical experience instead of just theory like other subjects in medical education. Recommendation from previous students received 14% of the participants. About 11% of the students attended the course because they were convinced that music therapy is calming, enables communication and can support healing. Another 11% found a course of five consecutive days very useful. About 6% of the students were interested in music therapy in the Neonatal Intensive Care Unit (Figure 3).

### Expectations of the Elective Course Music Therapy

**Figure 4.**
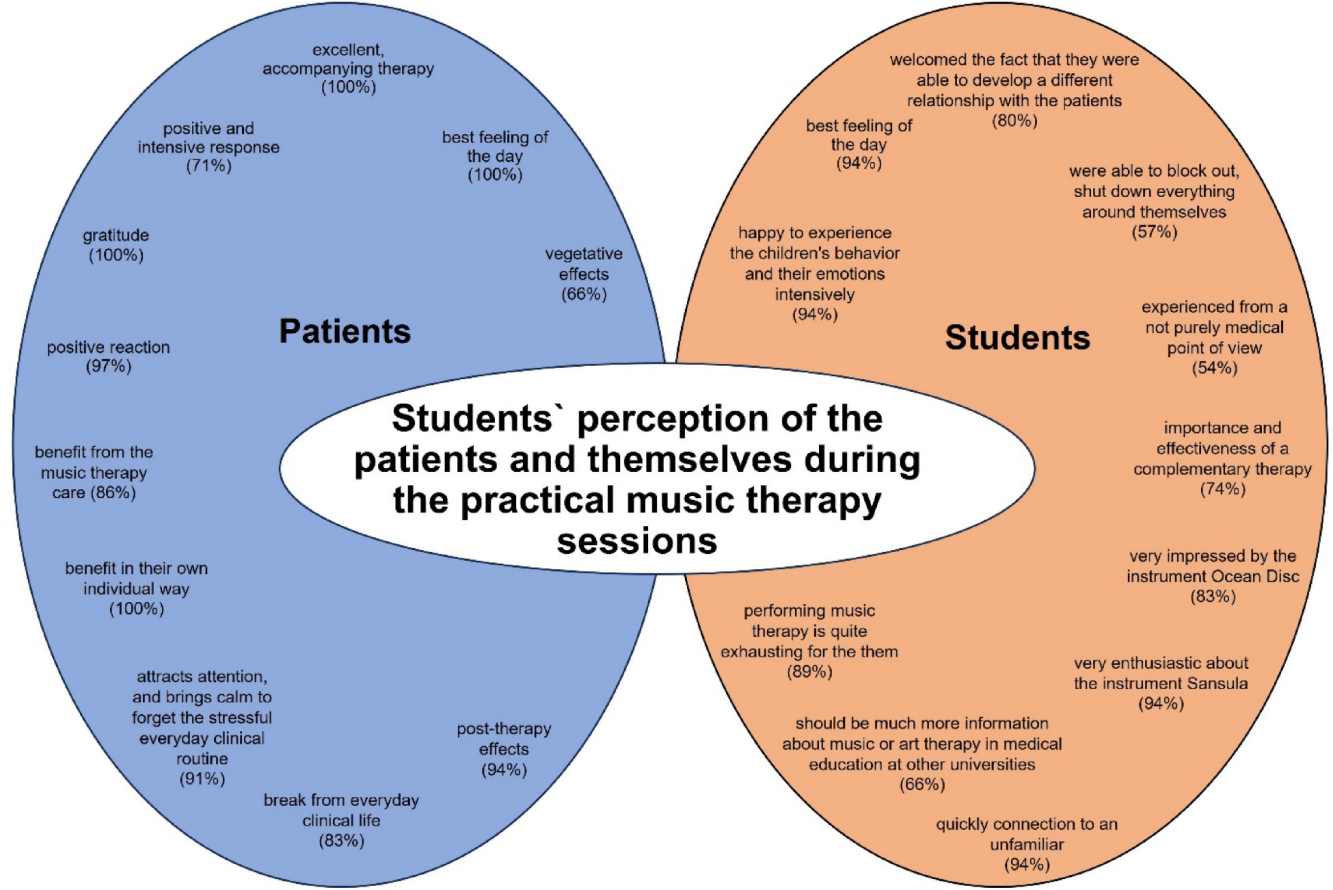
Students` perceptions of the patients and themselves during the practical music therapy sessions of the elective course *Music therapy in pediatrics (children's hospital and neonatology)*.

When asked whether the subject of music therapy corresponded to the ideas and wishes of the students, 91% of the students fully agreed. The remaining 9% replied that music therapy had even exceeded their expectations. About 40% of the students stated the fact that the subject was very practice oriented. The diversity and practical structure were perceived positively. In the opinion of the students, working independently and being involved was a great added value of the music therapy course. Some students described that they were able to gain valuable experience. An additional aspect mentioned of the students was that it was great to work with the children and to get an insight into the field of music therapy with children of different ages and clinical diagnoses, which they never had have before during their “normal” medical education. Some students appreciated the balance and combination of theoretical introductions and presentation of study results with practical experience and independent work. One student was enthusiastic to see the joy that music therapy brought to the children and their families (Figure 4).

### Perceptions During the Practical Part

Another part of the questionnaire was that the students were asked to describe and evaluate their perceptions of the music therapy sessions where they were present or where they directed together with the lecturer.

Music therapy was perceived as an excellent, accompanying therapy for both, sick children, and their parents by all students (100%). The students stated that in a stressful environment with negative emotions associated with doctors, medications and treatments, music therapy exuded joy and variety and brought out the best feeling of the day for both the patients (100%) and the students (94%). The children and their needs are the focus during a music therapy session. The child can choose which instruments it would like to play from those provided by the music therapist. In addition, the child is free to choose the volume and the tempo of the improvised music. About 71% of the students described that they observed a surprisingly positive and intensive response in the children during the therapy. Therapy success was visible in extremely different patients. Nearly all the students (97%) could clearly see how positively the children reacted to the music therapy. The concept was perceived as very convincing, and the care of the children as a real benefit in everyday hospital life. The students noted (86%) that each child showed her/his individual preferences and reactions, but all children benefited from the music therapy care. Some children became more alert, more communicative and less shy. Other children calmed down and fell asleep. The music is independent of age, connects, attracts attention, and brings calm to forget the stressful everyday clinical routine for the moment of music therapy described 91% of the students. About 94% of the students were amazed how quickly they could establish a connection to an unfamiliar person with the music. Music therapy is a distraction and a from everyday clinical life for both the children and the parents, 83% of the students observed. The students (57%) described how they were able to block out, shut down everything around themselves and fully concentrate on the children and on the music. All students stated that they were able to observe that all patients benefited from music therapy in their own individual way and with their individual needs and aims. Each session was unique, even with the same child on different days. About 83% of the students were very impressed by the instrument Ocean Disc. They expressed that they had not thought that the instrument would be so popular for the children. The students (94%) were also very enthusiastic about the instrument Sansula with the space-filling, long-lasting and soft sound.

Regarding the music therapy with preterm infants, 94% of the students were surprised to already observe so many reactions in the infants and to note post-therapy effects very well. Perceiving the smallest movements and changes in vital signs on preterm infants and reacting actively to them required a high degree of concentration and attention, which made the students realize how exhausting a day of practical work was for them. The students (89%) expressed that it should not be underestimated that therapist-directed music therapy is quite exhausting for the therapist.

All students could feel the gratitude of the children and parents and were fascinated how much value it had to inspire the children with music and how the children's illness was pushed into the background.

Experiencing and looking to the children and their parents from a different point of view, and not only from a medical perspective, was an experience the students had never done before during their medical education. They (94%) were happy to experience the children's behavior and their emotions intensively. About 66% of the students described that they found it amazing that music therapy also has effects in medical aspects, so that the effects can also be seen objectively watching the vital signs on the monitor. They were enthusiastic about the studies and results that are being carried out at the University Hospital in Essen.

54% of the students described that they experienced from a not purely medical point of view how valuable moments like those in the music therapy sessions can be for chronically ill children and preterm infants and their parents. Being able to experience the importance and effectiveness of a complementary therapy such as music therapy was an added value for the students (74%) during their medical education. The students (80%) welcomed the fact that they were able to develop a different relationship with the patients and their caregivers in the elective course music therapy compared to the clinical traineeships.

66% of the students suggested that there should be much more information about music or art therapy in medical education at other universities because the positive effect of creative support is often still underestimated.

### Experiences and Impressions for the Students’ Future

The students (91%) were fascinated that they could make music together without having any prior knowledge of an instrument. The professionalism aspect was completely irrelevant, which took away the pressure of having to have some skills. Music therapy does not require one to be able to sing or play an instrument, but to express oneself and communicate by making music. All students observed that it helps patients and parents when complementary therapies to the conventional medicine are offered. About 54% of the students reported that it is important that the ill children also have good experiences and the opportunity to express themselves with music and instruments and to relax during their hospital stay.

At least for the moment of the therapy session, a safe environment is promoted for the children. The importance of complementary forms of therapy to the conventional medicine has become clear for all students. About 69% of the students realized how sensitive younger children and preterm infants are to all kinds of sounds and how much influence music can have on the child's condition and well-being. Therefore, all students recognized the importance of paying more attention to background noises when working with the patients to create a more pleasant atmosphere. The students (51%) expressed that they experienced music as another language. With music therapy, the students (43%) wanted to give the patients the opportunity to experience their own self-determination, because usually patients are completely determined by others in everyday clinical life. The students also stated (60%) that during the elective course of music therapy they realized how important music is for them. The students (77%) emphasized that they take for their future to perceive the patient and its family independently of medical data, diagnoses, and contexts. They (94%) will recommend that patients and parents use complementary therapies such as music therapy. Music therapy offers many opportunities that are still not seen enough in everyday clinical practice. The students (86%) assessed music therapy as an area in need of support that still receives too little attention. Music is associated with direct interaction and, apart from frequencies, it has interpersonal aspects that improve the relationship between therapist, parents, and children. All students expressed that in the future they will not only want to see medicine and the patient as a medical case, but also want to observe which forms of therapy can support the patients on an emotional level. The students want to incorporate alternative therapies and supportive procedures like music therapy to show colleagues. The students (74%) emphasized that they realized once again that they should always remain open to new things. Music therapy should be further developed and established. The students (77%) perceived music therapy as an interesting area of ​​research and showed great interest in participating in further studies of music therapy.

All students praised the fact that they were able to gain experience in the special elective course that they would otherwise not be able to acquire in medical education.

### EVALuna Online Evaluation

Mean scores for responses in the EVALuna online evaluation were high for all questions, reflecting an overall high satisfaction with the course (Table 2).

**Table 2.** Mean satisfaction of the students with the elective course music therapy in medical education at University Duisburg-Essen.

	**Mean (Range)**
Overall, how satisfied are you with this course? n = 21	6.43 (4–7)
How satisfied are you with the performance of the lecturer? n = 21	6.71 (5–7)
How satisfied are you with the exam situation of this course? n = 20	6.45 (3–7)
How satisfied are you with the preparation for the exam during this course? n = 19	6.68 (5–7)
How satisfied are you with the organization of this course? n = 21	6.24 (3–7)
How satisfied are you with the content of this course? n = 21	6.33 (4–7)
How satisfied are you with the general conditions of this course (rooms, technical equipment, etc)? n = 19	6.37 (5–7)
How satisfied are you with the tools for the preparation and follow-up of this course (script, slides, etc)? n = 19	6.21 (3–7)
How do you rate your subjective increase in knowledge through this course? n = 21	6.14 (4–7)

The highest values we found were in the performance of the lecturer and the preparation for the exam during this course (Table 2).

## Discussion

This study supports that music therapy, as a supplementary form of therapy in routine clinical care, is an important topic for medical education. The students’ answers show a high level of interest in the field of music therapy. The positive evaluation of the course indicates that the students were able to get a deep experience with the method of the additional kind of therapy. The answers reflected that the students could not only achieve knowledge in music therapy according to the cognitive learning objectives, but especially gained positive experiences with the use and the efficacy of music therapy, in accordance with the affective and psychomotor (skill-oriented) learning aims. The results and positive effects presented here show how important it is to offer a course of music therapy in medical education. Physical, mental, and social well-being are important components and goals of a holistic health care.^38,39^ The psychological aspect is just as important in healing and recovery as the medical aspect and influences the effectiveness of therapy.^40^ Music therapy has been observed as an effective adjuvant treatment for patients, both children and adults, with chronic diseases as well as for those with various types of disabilities.^17^

Effective patient involvement in clinical practice can lead to higher patient satisfaction and better health outcomes and is essential to the delivery of patient-centered care.^41^ With growing pressure on acute healthcare systems and limited time resources of the medical staff,^42^ it is increasingly important for healthcare providers to have additional therapies such as music therapy.^43^

The self-esteem, which decreases due to the heteronomy in everyday clinical practice, can be strengthened by music therapy.^44,45^ Highly self-efficacious individuals choose more ambitious goals, put more effort into attaining them, and feel more capable. Self-efficacy beliefs influence people's feelings, thoughts, and actions. Therefore, the construct of self-efficacy has become an indispensable part of most theories of health behavior change.^46^

Several scientific findings show that in medical care the patients are seen more as their disease(s) and diagnosis than as individuals.^47^ An interdisciplinary work of physicians and therapists can treat and support the patients individually from different perspectives and improve patient outcomes.^48^ To fully consider the preferences, needs and values ​​of patients, a patient-centred care should be implemented.^49^

Individual music therapy is a positive, supportive offer for the patients and their caregivers and complements the patient-centred care. The therapy promotes relaxation and has a positive impact for the parent-child interaction^50^ and on multiple symptoms.^6^ The quality of life can be improved.^50^ According to parents’ observations, music therapy is an effectively integrated kind of complementary therapies in the clinical setting and can support the children during their hospital stay.^51^

Our study has several limitations. The music therapy course at the University of Duisburg-Essen is established as an elective course. Therefore, it is likely that only motivated and interested students participated in the subject. Further, the students experienced the various areas of application of music therapy in the theoretical part, but they only directed the music therapy practically in pediatrics and neonatology, not in other medical disciplines. One further limitation is that no sample size analysis was calculated for this study and the validity of our questionnaire was not tested on a randomized study.

Only musician medicine i.e. therapy for specific diseases in musicians, is offered in medical studies at the Medical Faculty of the University of Freiburg. This is unique nationwide for medical students in Freiburg because the Institute for Musicians’ Medicine is part of the Medical Faculty in Freiburg and can therefore train medical students as part of their studies.^52^

## Conclusions

The music therapy course has been successfully established in medical education at the University Duisburg-Essen and the topic is well suited to demonstrate students the importance of complementary therapies and holistic care for patients. Regardless of the medical specialty, complementary therapies such as music therapy, art therapy, drama therapy or dance therapy should be established in medical care and offered in medical curricula.

## Supplemental Material

sj-docx-1-mde-10.1177_23821205241234537 - Supplemental material for Music Therapy as a Topic in Medical Education: Course Concept and Student Evaluation of an Elective Course for Medical Students Supplemental material, sj-docx-1-mde-10.1177_23821205241234537 for Music Therapy as a Topic in Medical Education: Course Concept and Student Evaluation of an Elective Course for Medical Students by Susann Kobus, Ursula Felderhoff-Mueser, Elke Lainka, Joachim Fandrey and Sven Benson in Journal of Medical Education and Curricular Development

sj-docx-2-mde-10.1177_23821205241234537 - Supplemental material for Music Therapy as a Topic in Medical Education: Course Concept and Student Evaluation of an Elective Course for Medical Students Supplemental material, sj-docx-2-mde-10.1177_23821205241234537 for Music Therapy as a Topic in Medical Education: Course Concept and Student Evaluation of an Elective Course for Medical Students by Susann Kobus, Ursula Felderhoff-Mueser, Elke Lainka, Joachim Fandrey and Sven Benson in Journal of Medical Education and Curricular Development
