# Supplementary material for: Music Therapy as a Topic in Medical Education: Course Concept and Student Evaluation of an Elective Course for Medical Students
Source: J Med Educ Curric Dev. 2024 Feb 22;11:23821205241234537. doi: 10.1177/23821205241234537 (PMC10894546; doi:10.1177/23821205241234537)
Supplement: sj-docx-1-mde-10.1177_23821205241234537 - Supplemental material for Music Therapy as a Topic in Medical Education: Course Concept and Student Evaluation of an Elective Course for Medical Students [file sj-docx-1-mde-10.1177_23821205241234537.docx]

**Music therapy in pediatrics (children's hospital and neonatology)**

1. Why did you decide to take part at the course Music therapy in pediatrics (children's hospital and neonatology)?

2. Did the course fulfil your expectations and wishes?

3.  Describe and evaluate your perceptions that you made during the music therapy course in the children's hospital and neonatology!

4. What experiences and impressions will you take with you for your future?
