# Supplementary material for: Music Therapy as a Topic in Medical Education: Course Concept and Student Evaluation of an Elective Course for Medical Students
Source: J Med Educ Curric Dev. 2024 Feb 22;11:23821205241234537. doi: 10.1177/23821205241234537 (PMC10894546; doi:10.1177/23821205241234537)
Supplement: sj-docx-2-mde-10.1177_23821205241234537 - Supplemental material for Music Therapy as a Topic in Medical Education: Course Concept and Student Evaluation of an Elective Course for Medical Students [file sj-docx-2-mde-10.1177_23821205241234537.docx]

**EVALuna online evaluation tool**

Music therapy in pediatrics (children's hospital and neonatology)

|  | 1  Absolutely unsatisfied | 2  Mostly unsatisfied | 3  Somewhat unsatisfied | 4  neither satisfied or unsatisfied | 5  Somewhat satisfied | 6  Mostly satisfied | 7  Absolutely satisfied |
| --- | --- | --- | --- | --- | --- | --- | --- |
| 1. Overall, how satisfied are you with this course? | O | O | O | O | O | O | O |
| 2. How satisfied are you with the performance of the lecturers? | O | O | O | O | O | O | O |
| 3. How satisfied are you with the exam situation of this course? | O | O | O | O | O | O | O |
| 4. How satisfied are you with the preparation for the exam during this course? | O | O | O | O | O | O | O |
| 5. How satisfied are you with the organization of this course? | O | O | O | O | O | O | O |
| 6. How satisfied are you with the content of this course? | O | O | O | O | O | O | O |
| 7. How satisfied are you with the general conditions of this course (rooms, technical equipment, etc.)? | O | O | O | O | O | O | O |
| 8. How satisfied are you with the tools for the preparation and follow-up of this course (script, slides, etc.)? | O | O | O | O | O | O | O |
| 9. How do you rate your subjective increase in knowledge through this course? | O | O | O | O | O | O | O |
